# Influence of Dimerization of Lipopeptide Laur-Orn-Orn-Cys–NH_2_ and an *N*-terminal Peptide of Human Lactoferricin on Biological Activity

**DOI:** 10.1007/s10989-014-9423-y

**Published:** 2014-08-21

**Authors:** Elżbieta Kamysz, Emilia Sikorska, Małgorzata Dawgul, Rafał Tyszkowski, Wojciech Kamysz

**Affiliations:** 1 Faculty of Chemistry, University of Gdańsk, Wita Stwosza 63, 80-308 Gdańsk, Poland; 2 Faculty of Pharmacy, Medical University of Gdańsk, Al. Gen. Hallera 107, 80-416 Gdańsk, Poland

**Keywords:** Antimicrobial activity, Circular dichroism, Haemolytic activity, Lactoferricin, Lactoferrin, Lipopeptide

## Abstract

Lactoferrin (LF) is a naturally occurring antimicrobial peptide that is cleaved by pepsin to lactoferricin (LFcin). LFcin has an enhanced antimicrobial activity as compared to that of LF. Recently several hetero- and homodimeric antimicrobial peptides stabilized by a single disulfide bond linking linear polypeptide chains have been discovered. We have demonstrated that the S–S bond heterodimerization of lipopeptide Laur-Orn-Orn-Cys–NH_2_ (peptide III) and the synthetic *N*-terminal peptide of human lactoferricin (peptide I) yields a dimer (peptide V), which is almost as microbiologically active as the more active monomer and at the same time it is much less toxic. Furthermore, it has been found that the S–S bond homodimerization of both peptide I and peptide III did not affect antimicrobial and haemolytic activity of the compounds. The homo- and heterodimerization of peptides I and III resulted in either reduction or loss of antifungal activity. This work suggests that heterodimerization of antimicrobial lipopeptides via intermolecular disulfide bond might be a powerful modification deserving consideration in the design of antimicrobial peptides.

## Introduction

**Fig. 1:**
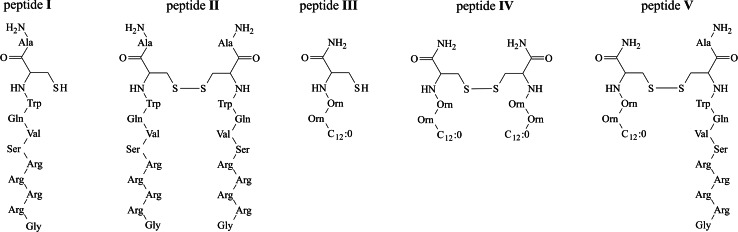
The primary structures of the peptides studied

Antimicrobial peptides are widely distributed in nature to counteract the action of microbes. However, an increasing number of bacterial strains resistant to conventional antibiotics justifies an intense search for new antimicrobial agents. One example of naturally occurring antimicrobial peptides is lactoferrin (LF), a multifunctional iron-binding glycoprotein originally discovered in bovine milk (Sorenson and Sorenson 1939) and later in exocrine secretions of mammals and in granules and neutrophils during inflammatory responses (Masson et al. 1966; Masson et al. 1969). Pepsin cleavage of LF leads to lactoferricin (LFcin) that has an enhanced antimicrobial activity as compared to that of LF (Bellamy et al. 1992; Gifford et al. 2005). LFcin inhibits growth of a diverse range of microorganisms, such as Gram-negative bacteria, Gram-positive bacteria, yeast, filamentous fungi, and parasitic protozoa, including some antibiotic-resistant pathogens (Wakabayashi et al. 2003). A synthetic peptide corresponding to the first 11 *N*-terminal amino acids of human LFcin and LF, referred to as hLF (1–11) has been shown to be highly microbicidal towards various strains of drug-resistant bacteria (*Staphylococcus aureus, Listeria monocytogenes, Klebsiella* *pneumoniae, Escherichia coli, Acinetobacter baumannii*) and fungi (*Candida albicans, Aspergillus fumigates*) (Dijkshoorn et al. 2004). One of the strategies to improve antimicrobial activity may be attachment of a hydrophobic chain that can compensate for lack of hydrophobicity (Avrahami and Shai 2004). *N*-acylation of derivatives based on a sequence of bovine lactoferricin B (amino acids 17–41) resulted in a higher antibacterial activity (Wakabayashi et al. 1999; Strøm et al. 2002). However, such a strategy can also result in a higher toxicity towards eukaryotic cells, thus resulting in loss of the target cell selectivity (Zweytick et al. 2006; Blondelle et al. 1999; Avrahami and Shai 2004). We decided to study the effect of intermolecular disulfide bond dimerization of lipopeptide Laur-Orn-Orn-Cys–NH_2_ (peptide **III**) and the synthetic *N*-terminal peptide of human LF, hLF(1–11)–NH_2_ (peptide **I**) on antimicrobial and haemolytic activity as well as on conformational changes. Creating homo- or heterodimers by intermolecular disulfide bonds confer the following properties that may enhance therapeutic potential of antimicrobial peptides: stability, efficiency, toxicity, maintenance of activity in high salt concentrations and under physiological conditions, and overcoming bacterial resistance. The primary structures of all the peptides studied are displayed in Fig. 1. A lipopeptide with lauric acid was selected based on previous studies demonstrating that insertion of a 12 hydrocarbon chain into the *C*-terminal or *N*-terminal region of LFcin 21–31 leads to enhancement of antibacterial activity (Majerle et al. 2003; Andrä et al. 2005). The conformations of peptides was determined in two micellar systems, dodecylphosphocholine (DPC) and sodium dodecyl sulfate (SDS), to examine the effect of headgroup charge on the peptide structure. The former provides a zwitterionic, whereas the latter a negatively charged surface on the micelles, which mimics biological membranes of the vertebrates and microorganisms, respectively (Scrima et al. 2007; Jaroniec et al. 2005).

## Materials and Methods

### Peptide Synthesis

All peptides were synthesised by the solid-phase method on Tentagel S Ram Fmoc resin (0.23 mmol/g, RAAP Polymere, Germany) using Fmoc chemistry (Chan and White 2000). *N*-*α*-Fmoc-protected amino acids and ethyl 2-cyano-(hydroxyimino) acetate (Oxyma Pure) were purchased from Iris Biotech GmbH (Germany); piperidine, chloranil, lauric acid, iodine, diisopropylethylamine (DIEA), triisopropylsilane (TIS) and *O*-(benzotriazol-1-yl)-1,1,3,3-tetramethyluronium tetrafluoroborate (TBTU), acetonitrile (ACN), *N,N*-dimethylformamide (DMF), dichloromethane (DCM), methanol, acetic acid, and diethyl ether were from Sigma-Aldrich (Poland). The following amino acids side chain protecting groups were used: Trt (Gln, Cys), tBu (Ser), Boc (Orn, Trp), Pbf (Arg). Peptides’ chains were elongated in the consecutive cycles of deprotection and coupling. Deprotection of the Fmoc group was carried out with a 20 % piperidine in DMF. The coupling reactions were carried out with the protected amino acid (Fmoc-AA) dissolved in DMF/DCM using Oxyma Pure and DIC for 2 h (Fmoc-AA:Oxyma Pure:DIC, 1:1:1). The completeness of each coupling step was monitored by the chloranil test. If positive, the coupling reactions were repeated using TBTU with addition of Oxyma Pure in the presence of DIEA for 2 h (Fmoc-AA:TBTU:OxymaPure:DIEA, 1:1:1:2). Lauric acid was coupled in the same manner as Fmoc-AA. After the syntheses have been completed, the peptidyl resins were dried under vacuum. The final peptides were cleaved from the resin along with the side chain deprotection in a one-step procedure using a mixture of TFA/phenol/TIS/H_2_O (90:2.5:2.5:5, v/v/v/v) for 2 h. The cleaved peptides were precipitated with cold diethyl ether and lyophilised.

Peptide **I** and peptide **III** were purified using the RP-HPLC on a Phenomenex Columbus C18 column (10 × 250 mm, 100 Å pore size, 5 μm particle size) with 0.1 % aqueous TFA [A] and 0.1 % TFA in ACN [B] solvent systems. The eluates were fractionated and analysed by analytical RP-HPLC. The purity of the peptides was checked on a Varian ProStar HPLC system controlled by a Galaxie Chromatography Data System with Phenomenex Onyx Monolithic C18 column (100 × 4.6 mm) using 0.1 % aqueous TFA [A] and 0.1 % TFA in ACN [B] solvent systems. Fractions containing the pure peptides (>98 %) were pooled and lyophilized.

In the next step, the Cys-linked dimers (peptide**s** **II**, **IV**, **V**) were prepared by dissolving the monomers (peptides **I** and **III** in 20 % (v/v) AcOH (peptide concentration: 1 mg/mL). The free thiol groups of the peptides were oxidized by adding dropwise a 0.01 M solution of iodine in methanol. The solution was stirred at room temperature. The formation of dimers was checked by comparison of analytical RP-HPLC profiles between monomer and dimer and confirmed by MALDI-TOF MS. Purification of dimeric forms was performed in the same manner as that of the monomeric forms. Physicochemical characteristics of all synthesized peptides are presented in Table 1.

**Table 1:** Physicochemical characteristics of the peptides I–V

Symbol	Formula	HPLC R_t_ (min)^*^	calculated monoisotopic mass	[M + H]^+^ found
Peptide **I**	C_56_H_95_N_26_O_13_S_1_	2.9	1,371.7	1,372.6
Peptide **II**	C_112_H_188_N_26_O_13_S_1_	4.3	2,741.4	2,742.4
Peptide **III**	C_25_H_50_N_6_O_4_S_1_	11.4	530.3	531.4
Peptide **IV**	C_50_H_98_N_12_O_8_S_2_	9.6	1,058.6	1,059.4
Peptide **V**	C_81_H_143_N_32_O_17_S_2_	6.8	1,900.0	1,901.1

^*^linear gradient from 10–80 % of [B] for 20 min, Phenomenex Onyx Monolithic C18 column (100 × 4.6 mm) using a flow rate of 3 mL/min and with the following solvent systems: 0.1 % aqueous TFA [A], 0.1 % TFA in acetonitrile [B]

### Antimicrobial Susceptibility Testing

The reference strains were supplied by the Polish Collection of Microorganisms (Polish Academy of Sciences, Institute of Immunology and Experimental Therapy, Wroclaw, Poland). Minimal inhibitory concentration (MIC) was determined by the microbroth dilution method outlined by the National Committee for Clinical Laboratory Standards (2003). The following microbial strains were tested: Gram-positive: *Rhodococcus equi* ATCC 6939, *Staphylococcus epidermidis, S. aureus* ATCC 6538, *Streptococcus pyogenes and Enterococcus faecalis*; Gram-negative: *E. coli* ATCC 8739, *Pseudomonas aeruginosa* and fungi: *C. albicans* ATCC10231. Bacteria were suspended in Mueller–Hinton II broth (Sigma-Aldrich) at initial inocula of 5 × 10^5^ cfu **(**colony-forming units)/mL, while the Sabouraud 5 % dextrose broth of a pH of 7.4 (Sigma-Aldrich) was used for fungi (at initial inoculum of 5 × 10^3^ cfu/mL). Microbial cells in polystyrene 96-well plates were exposed to the peptides at adequate concentrations (range 1–512 μg/mL) for 18 h at 37 °C (bacterial strains) or for 48 h at 25 °C (fungi). MIC was taken as the lowest drug concentration at which observable growth was inhibited. Experiments were performed in triplicate on three different days.

### Haemolysis Assay

The haemolytic activity of the peptides was tested against human red blood cells, essentially as described previously (Kamysz et al. 2007). Fresh human blood was collected into tubes containing EDTA as anticoagulant. Red blood cells were separated by centrifugation at 500×*g* for 12 min at room temperature and the plasma was aspirated. Erythrocytes were washed three times with PBS, separated by centrifugation (500×*g*, 12 min) and resuspended in PBS. Peptides I–V were serially diluted in PBS (range 1–512 μg/mL) and 50 μL was added to 50 μL of the erythrocyte suspension. The final concentration of red blood cells was 4 % [v/v]. The results were assessed visually after incubation for 2 h at 37 °C. As a positive control, 1 % Triton was used, whereas PBS served as a negative control.

### Circular Dichroism (CD) Measurements

CD spectra were recorded on peptide samples in a following solutions: 10 mM phosphate buffer at pH 3, 5 and 7 without and with addition of DPC or SDS in micellar concentrations. The CD spectra were collected at 25 °C. The measurements were conducted on 0.1 mg/mL peptide solutions. A Jasco J-815 spectropolarimeter with a 2 cm/min scan speed was used (Physicochemical Laboratories, Faculty of Chemistry, University of Gdansk, Poland). Although all the spectra were recorded over the 195–260 nm range, in same cases their fragments below ~200 nm were not reliable due to strong interferences of the solvent at those wavelengths. The signal/noise ratio was increased by acquiring each spectrum over an average of three scans. Finally, each spectrum was corrected by subtracting the background from the sample spectrum and plotted as mean molar ellipticity Θ (degree × cm^2^/dmol) versus wavelength λ (nm). The content of the secondary structure was calculated from the spectra using a CONTINLL method (Sreerama and Woody 2000).

## Results and Discussion

### Peptide Synthesis

We synthesized five peptides to estimate their antimicrobial and haemolytic activity and to perform conformational analysis by circular dichroism. Physicochemical characteristics of all the synthesized peptides are presented in Table 1.

### Antimicrobial and Haemolytic Activity

Biological activity of the synthesized peptides was tested against Gram-positive bacteria, Gram-negative bacteria and fungi (Table 2). All the test organisms are human pathogens. In this study the most effective antimicrobial compound was peptide **III** (Laur-Orn-Orn-Cys–NH_2_), but it was also the most haemolytic one (Table 3). Laur-Orn-Orn-Cys–NH_2_ displayed antimicrobial activity against *S. aureus* comparable to that of Pal-Lys-Lys–NH_2_ and Pal-Arg-Arg–NH_2_ (Dawgul et al. 2012). Homodimeric forms of both peptide **I** (hLF(1–11)–NH_2_) and peptide **III** exerted the same antimicrobial and haemolytic activity as did their monomeric forms. Interestingly, peptide **V** (a heterodimer of peptide **I** and **III**) was almost as active against Gram-positive and Gram-negative bacteria as peptide **III** but it was much less haemolytic than peptide **III**. Peptide **III** showed only for *E. faecalis* and *E. coli* a four-times higher MIC than peptide **V**. Furthermore, peptide **III** exhibited a stronger antimicrobial activity than peptide **I**. The largest difference in the antimicrobial activity was noticed with *S. aureus*. In this case peptide **III** displayed antimicrobial activity up to four orders of magnitude stronger than did peptide **I**. Antifungal activity of peptide **V** remained unnoticed up to a concentration of 512 µg/mL. In our experiments, both homo- and heterodimerization of peptide**s** **I** and **III** resulted in either reduction or loss of antifungal activity. Recently, a number of natural and synthetic molecules with intermolecular disulfide bonds creating homo- and heterodimers have been reported (Campopiano et al. 2004; Dempsey et al. 2003; Lee et al. 2001; Yomogida et al. 1996; Jang et al. 2003; Zhu and Shin 2009; Cirioni et al. 2011). However enhancement of antimicrobial activity was not always accompanied by a concomitant decrease in haemolytic activity. For instance, a disulfide-dimerized cysteine substitution analogue of magainin-2 amide showed an enhanced antimicrobial activity against the Gram-negative bacteria as compared to that of the monomeric peptide. Unfortunately, in this case the disulfide dimerization enhanced haemolytic activity (Dempsey et al. 2003). Lee et al. (2001) reported a homodimer dicynthaurin isolated from haemocytes of the solitary tunicate, *Halocynthia aurantium*. The monomeric and dimeric forms were active against Gram-negative and Gram-positive bacteria, but not against *C. albicans*. Both forms showed equivalent activity on a weight basis. The dimer was more haemolytic than the monomer. The dimeric form (di-Tat(W)–C) of the Tat(W)–C (48–60) analogue, [Tat(W): GRKKRRQRRRPWQ–NH_**2**_], exhibited almost similar antimicrobial activity against the six bacterial strains tested as that of the monomeric form. The peptides did not cause haemolysis even at the highest concentration tested (200 μM) (Zhu and Shin 2009). A homodimer of lipopeptide Laur-Cys-Lys-Lys–NH_2_ was synergistic and prevent**s** daptomycin resistance to severe enterococcal infections (Cirioni et al. 2011). These results suggest that the potency and selectivity of antimicrobial peptides can be modulated with heterodimerization of lipopeptides via intermolecular disulfide bond. Moreover, dimerization increases stability of cysteine-containing peptides which must be handled with care to prevent side reactions because the thiol (–SH) containing side chain of cysteine is susceptible to oxidization.

**Table 2:** Antimicrobial activity of the peptides I–V

Organism	Antimicrobial activity (µg/mL)				
	Peptide **I**	Peptide **II**	Peptide **III**	Peptide **IV**	Peptide **V**
Gram-positive bacteria					
*Enterococcus faecalis*	256	256	2	4	32
*Rhodococcus equi*	16	8	2	1	2
*Staphylococcus aureus*	64	64	2	2	4
*Staphylococcus epidermidis*	2	2	2	2	1
*Streptococcus pyogenes*	2	1	4	2	2
Gram-negative bacteria					
*Escherichia coli*	512	512	8	8	128
*Pseudomonas aeruginosa*	32	32	8	4	8
Fungi					
*Candida albicans*	128	256	128	>512	>512

**Table 3:** Haemolytic activity (µg/mL) of the peptides I–V

Cells	Peptide **I**	Peptide **II**	Peptide **III**	Peptide **IV**	Peptide **V**
Human red blood cells	>512	>512	16	16	128

### CD

The CD spectra of those peptides whose solution state is characterized by an ensemble of conformers with different contents of secondary structures, represent obviously an average of the contributions from different conformers. Moreover, the spectra recorded in the presence of micelles provide a weighted average of the micelle-bound and free peptide conformations. This notwithstanding, the circular dichroism is a powerful method to track the peptide conformational changes upon modification of external conditions.

**Fig. 2:**
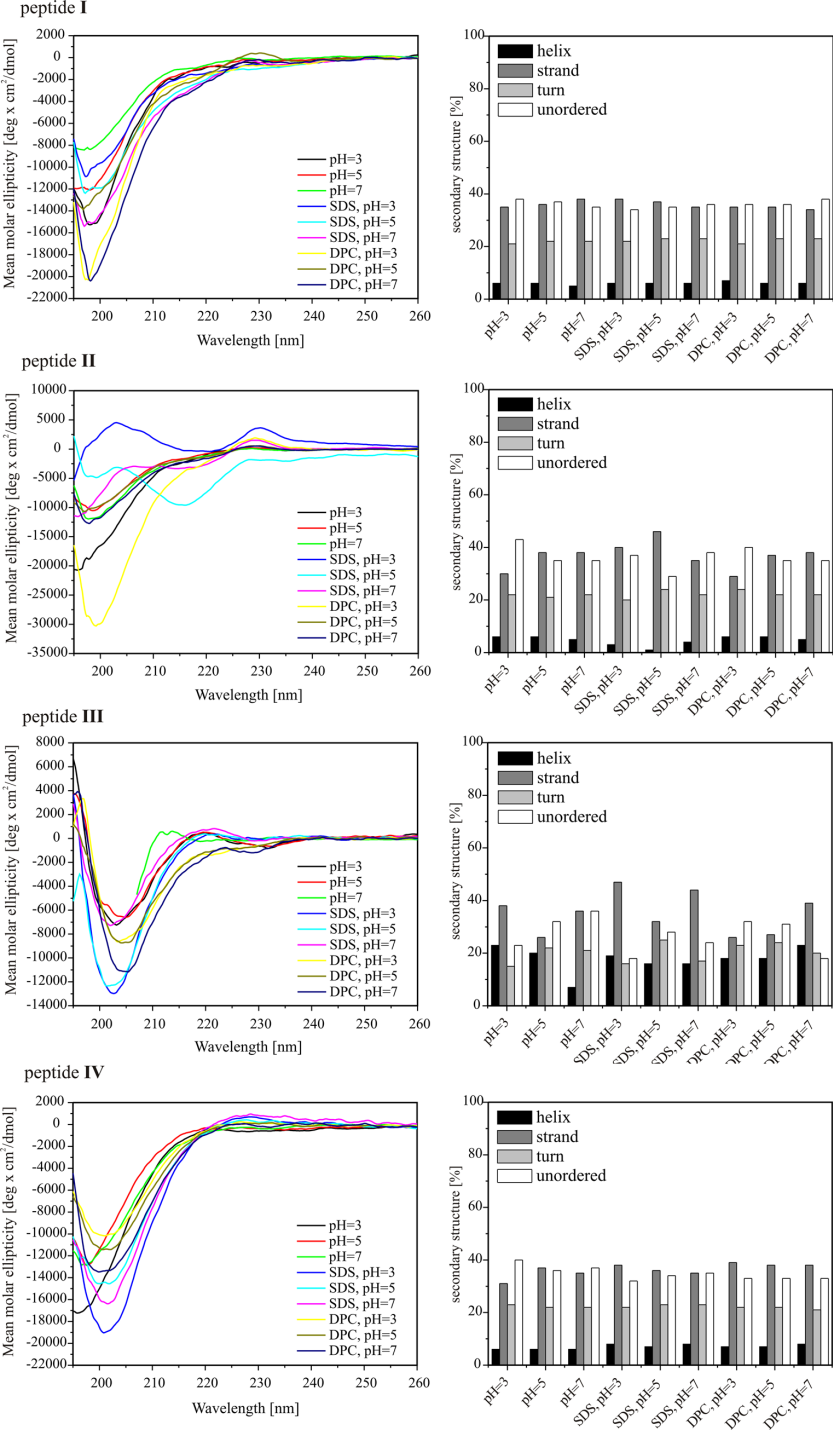

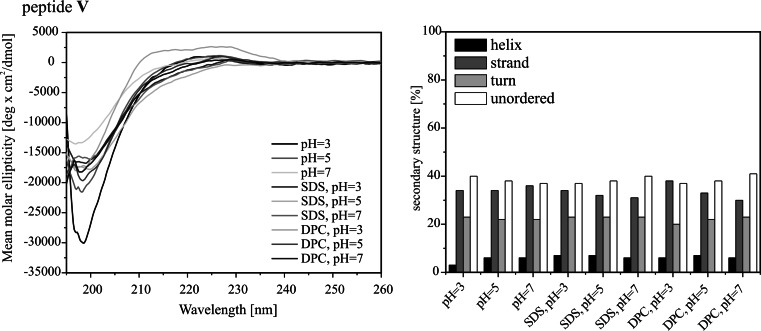
CD spectra (*left panel*) and CD data analysis via the CONTINLL algorithm (*right panel*) of the peptides studied under different experimental conditions

The CD spectra of the peptides studied are presented in Fig. 2. As seen, the spectra of peptide **I**, being a fragment of lactoferricin, are characterized by a deep negative minimum at 198 nm, due to π–π* transition of the amide groups under all experimental conditions. A quantitative CD analysis reveals that the conformation of peptide **I** is a mixture of an extended, turn and unordered structure and remains unchanged upon addition of detergents, DPC or SDS. These results are consistent with those obtained for lactoferricin (Hunter et al. 2005), where the *N*-terminus has been found not to be involved in any regular structure formation. With peptide **II**, being a dimer of peptide **I**, the SDS micelles at pH 3 and 5, induce noticeable changes in the shape of the CD spectra. These spectra display two bands: a minimum at 215 nm and a maximum at *circa* 200 nm, which show a clear preference for β-strand formation.

With peptides **III** and **IV**, the negative minimum due to π–π* transition of the amide groups is slightly red-shifted in comparison to that of the remaining peptides. However, in the case of the latter, such a change is observed only upon addition of detergent. In both cases, this finding suggests that the peptides are more structured than the remaining ones. In the case of peptide **III**, the deconvolution of the CD spectra reveals that the content of helical structure is more than twice higher than for the other peptides studied and represents an average 20 % of the entire conformation. However, taking into account the length of peptide **III**, it is more probable that the increase of helical structure content is the result of formation of βIII-turn, being a single unit of 3_10_-helix (Sudha et al. 1983; Ananthanarayanan and Brimble 1996). An exception is a sample placed in a detergent-free phosphate buffer solution at pH 7, where the helical structure content reaches only 7 %, similar to that of the other remaining peptides. With peptide **IV**, the disulfide-linkage does not predispose the peptide to helix-like conformation. It is likely that introduction of the disulfide bond in the *C*-terminus results in steric interactions, which makes difficult formation of a hydrogen bond between amide protons of the *C*-terminal Cys^4^ and carbonyl oxygen atom of lauric acid to close a 10-membered ring of βIII-turn. Consequently, peptide **IV** shows the tendency to adopt an extended conformation.

The CD spectra of peptide **V**, being a mixed dimer of peptides **I** and **III**, are characterized by a deep minimum at 198 nm similar to that of peptide **I**, a lactoferricin fragment. In addition, a quantitative analysis of the CD spectra confirms the conclusion that conformation of the mixed dimer is close to that adopted by peptide **I**, this indicating that just the lactoferricin fragment determines the overall structure of peptide **V**.

## Conclusion

In this paper, we have demonstrated that S–S bond heterodimerization of peptide**s** **I** and **III** yields dimer, which is almost as microbiologically active as the more active monomer and at the same time it is much less toxic. However, the S–S bond homodimerization both peptide**s** did not affect antimicrobial and haemolytic activities of the modified compounds. The homo- and heterodimerization of peptide**s** **I** and **III** resulted in either the reduction or loss of antifungal activity.

The results of analysis of the CD spectra indicate that the peptides studied do not adopt a well-defined stable structure. An exception may be peptide **III**, where a noticeable increase in the helix-like structure has been found as compared to that of the remaining peptides. It is likely that the peptide exhibits the tendency to create an amphipathic βIII-turn structure in which lauric acid and cysteine are buried in a micelle with ornithine residues present on opposite sides of the structure, which results in the most antimicrobial active compound. On the other hand, the same tendency is not retained in peptide **IV**, being a disulfide-linked homodimer of peptide **III**. Nonetheless, it exhibits a similar antimicrobial and haemolytic activity as that of the monomeric form. We suppose that peptide **IV**, by analogy to its monomeric form, possesses well-defined hydrophobic and charged surfaces, which enables its interactions with membrane.

In summary, the S–S bond dimerization may be applied to improve biological properties of novel antimicrobial agents that are effective in a variety of environments. Although much remains to be done, the results of this study suggest that heterodimerization of lipopeptides via intermolecular disulfide bond might be a powerful modification to deserve consideration in the design of antimicrobial peptides.
